# Screening for β-Thalassemia Trait in Pregnant Women With Microcytic Hypochromic Anemia and Its Impact on Pregnancy Outcomes

**DOI:** 10.7759/cureus.108206

**Published:** 2026-05-03

**Authors:** Dimple Bamrah, Ruchira Nautiyal, Mansi Kala, Kiran Bhat, Sumit Garg

**Affiliations:** 1 Department of Obstetrics and Gynaecology, Himalayan Institute of Medical Sciences, Dehradun, IND; 2 Department of Pathology, Himalayan Institute of Medical Sciences, Dehradun, IND; 3 Department of Biochemistry, Himalayan Institute of Medical Sciences, Dehradun, IND

**Keywords:** hematological indices, iron deficiency anemia, neonatal outcomes, pregnancy, β thalassemia trait

## Abstract

Background

Thalassemia is a common autosomal recessive hemoglobinopathy and a major public health concern in India. In pregnancy, β-thalassemia trait often presents as microcytic hypochromic anemia and may mimic iron deficiency anemia, leading to misdiagnosis and inappropriate management. Early and accurate differentiation is essential to prevent unnecessary iron therapy and reduce adverse maternal and neonatal outcomes. The study was designed to evaluate the prevalence, diagnostic differentiation, and pregnancy outcomes associated with β-thalassemia trait in this population.

Methods

This prospective observational study was conducted over a period of 12 months at a tertiary care center in North India. A total of 171 pregnant women with hemoglobin ≤11 g/dL and microcytic hypochromic anemia were enrolled. All participants underwent complete hemogram, peripheral smear examination, serum ferritin estimation, and calculation of discriminant indices, including the Mentzer index, the Shine and Lal index, and the RDW index. Patients with normal or elevated serum ferritin levels and indices suggestive of β-thalassemia underwent confirmatory testing using high-performance liquid chromatography and hemoglobin H preparation to rule out alpha thalassemia. Maternal outcomes such as mode of delivery, obstetric complications, and transfusion requirements, along with neonatal outcomes including birth weight, gestational age, Apgar score, and NICU admission, were recorded.

Results

Iron deficiency anemia was observed in 150 (87.7%) women, while β-thalassemia trait was identified in 21 (12.3%). Women with β-thalassemia trait had significantly lower hemoglobin levels (7.90 ± 1.36 vs 9.05 ± 0.93 g/dL), lower MCV and MCH values, and higher RBC counts (p = 0.001). Discriminant indices showed excellent diagnostic performance, with the Mentzer index, RDW index, and MCH demonstrating very high accuracy. Serum ferritin levels were <15 ng per ml in all iron deficiency anemia cases and >15 ng per ml in 95.2% of β-thalassemia cases (p = 0.001). Severe anemia was significantly more frequent in the β-thalassemia group (33.3% vs 1.3%) along with a higher requirement for packed red cell transfusion (42.9% vs 13.3%; p = 0.003). Obstetric complications such as severe preeclampsia, postpartum hemorrhage, and ICU admission were significantly more common in β-thalassemia trait. Neonatal outcomes were significantly worse, with higher rates of preterm birth (38.1% vs 4.1%), intrauterine growth restriction (47.6% vs 4.1%), low birth weight (61.9% vs 4.8%), and NICU admission (89.4% vs 15.8%) (p = 0.001).

Conclusion

β-thalassemia trait contributes significantly to anemia in pregnancy and is associated with increased maternal and neonatal morbidity. Simple hematological indices combined with serum ferritin estimation provide reliable and cost-effective screening tools for differentiating β-thalassemia trait from iron deficiency anemia. Early detection and appropriate management may help improve pregnancy outcomes.

## Introduction

Thalassemia is an autosomal recessive condition that causes impaired globin peptide chain synthesis during hemoglobin formation, impairing erythropoiesis and causing hemolysis and ultimately leading to anemia [1]. The hemoglobin molecule contains four globin subunits, two α (alpha) and two β (beta) chains, produced by their respective genes. Thalassemia is classified as α or β, depending on which globin gene is defective [2]. Two copies of a defective gene are required for thalassemia, an autosomal recessive condition; carriers, or heterozygotes, with one abnormal copy may not exhibit symptoms but are able to pass the abnormal gene on to their progeny. The amount of functional globin genes determines the severity of thalassemia. The loss of all four alpha globin genes (-/-) is known as α-thalassemia major. In β-thalassemia, carriers with one normal gene have mild anemia or no symptoms [3].

β-thalassemia is the most prevalent form of hemoglobinopathy observed in India, with a mean prevalence of 3.3% [4]. Worldwide, about 5.2% of individuals are carriers of clinically important hemoglobin variants, with the prevalence rising to over 7% among pregnant women. India bears a particularly high burden of thalassemia, often being referred to as its global epicenter, with estimates indicating that nearly one-eighth of all thalassemia carriers live in the country [5]. India accounts for the largest number of children with thalassemia major worldwide, with estimates ranging from 100,000 to 150,000 affected cases. Additionally, nearly 42 million individuals in the country are carriers of the thalassemia trait. It is estimated that about 10,000 to 15,000 infants are born with thalassemia major in India each year [6].

At present, bone marrow transplantation remains the only definitive curative option for thalassemia major. However, its application is limited by high costs, restricted access, and the scarcity of suitably matched human leukocyte antigen (HLA) donors, as well as the limited number of specialized transplant centers [7]. In India, the mean annual expenditure for the management of a patient with thalassemia is approximately INR 74,948, with reported costs ranging from INR 41,514 to INR 151,800. However, cost analyses indicate that the true annual expense of treatment, when calculated at prevailing market rates without government subsidies, may be as high as INR 167,750 per patient [8].

The best and most practical solution to this issue is preventive genetics (screening). The only effective strategy to deal with such a disease is a prospective preventive strategy that incorporates prenatal diagnosis, genetic counseling, mass screening, and public education. As a short-term program, an antenatal diagnosis is seen to be highly beneficial. This can entail identifying the carriers and providing a prenatal diagnosis for couples who are "at risk" of giving birth to children with thalassemia [9]. There are a number of screening techniques available for pregnant mothers, including peripheral blood smears, quantitative osmotic fragility testing, red cell indices assessment, and free red cell porphyrins. The hemoglobin A2 (HbA2) estimation test can confirm the β-thalassemia trait but needs advanced equipment and comes with a high learning curve, with involvement of high cost [10].

Since β-thalassemia minor is a heterozygous condition, patients are typically diagnosed with low mean cell volume, low mean corpuscular hemoglobin, raised HbA2, normal or elevated hemoglobin F (HbF), and moderate anemia (hemoglobin A level 1 or 2 g below the normal range). Women with thalassemia minor frequently experience more severe anemia during pregnancy, which is typically most noticeable in the later part of the second and early third trimesters [11].

Hematological problems in children have been linked to prematurity. A recognized consequence of β-thalassemia minor, early-term birth (37-38 weeks of gestation), has been demonstrated to independently correlate with the offspring's subsequent pediatric hematologic morbidity [12]. There is a need for oral iron therapy right from infancy through the reproductive years. Considering the risk of thalassemia overlapping with microcytic hypochromic on smear findings, many of the patients who do not respond adequately to oral hematinics remain undiagnosed.

With this background, and since there is a scarcity of research on thalassemia screening in pregnant women in the Uttarakhand region of India, the primary objective of this study was to determine the prevalence of β-thalassemia trait among pregnant women with microcytic hypochromic anemia. The secondary objectives were: (a) to evaluate the diagnostic performance of red cell indices and serum ferritin in differentiating β-thalassemia trait from iron deficiency anemia, and (b) to compare maternal and neonatal outcomes between the two groups.

## Materials and methods

Study design and setting

This prospective observational hospital-based study was conducted in the Department of Obstetrics and Gynaecology at Himalayan Institute of Medical Sciences, Swami Ram Nagar, Dehradun, after obtaining approval from the Institutional Ethics Committee (Ref No. SRHU/HIMS/ETHICS/2024/352). The study included pregnant women attending the antenatal clinic as well as those admitted to the Obstetrics ward during the study period. The total duration of the study was 12 months.

Sample size calculation

The required sample size was estimated using the formula $n = \frac{Z^2 \times P \times (1 - P)}{d^2}$, where n denotes the sample size, Z is the standard normal deviate for the chosen confidence level, P represents the anticipated prevalence, and d indicates the acceptable margin of error (precision). Based on a previous study by Kulkarni et. al. [9], the prevalence of β-thalassemia was taken as 8.5% among pregnant women. Taking a confidence level of 95% and a precision of 5%, the calculated sample size was 120.

Study population

Pregnant women with hemoglobin levels less than or equal to 11 g/dL with microcytic hypochromic anemia attending the antenatal clinic or admitted to the obstetrics ward were included in the study. Women who were unwilling to participate and those with known systemic illnesses such as chronic kidney disease, chronic liver disease, aplastic anemia, and malignancy were excluded. Relevant clinical details, including obstetric history and consanguinity, were recorded using a structured proforma through direct interview.

Laboratory evaluation and diagnostic protocol

Under aseptic precautions, 3 ml of venous blood was collected in plain and EDTA vacutainers from each participant. Samples were analyzed for complete hemogram, including red blood cell indices, using an automated hematology analyzer. Peripheral smear examination was performed in all cases with hemoglobin levels less than 11 g/dL. Patients with microcytic hypochromic anemia underwent further evaluation with serum ferritin estimation and calculation of red cell indices, including the Mentzer index [13], RDW index [14], and Shine and Lal index [15], to differentiate iron deficiency anemia from β-thalassemia trait. The Mentzer index was calculated as the ratio of mean corpuscular volume (MCV) to red blood cell (RBC) count, with a value less than 13 considered suggestive of β-thalassemia trait.

The Shine and Lal index was derived using the formula $\frac{MCV^2 \times MCH}{100}$, where MCH is the mean corpuscular hemoglobin, with values below 1530 indicating a likelihood of β-thalassemia trait. Similarly, the RDW index was calculated as MCV multiplied by red cell distribution width (RDW) divided by RBC count, and a value less than 220 was taken as suggestive of β-thalassemia trait. All participants meeting the inclusion criteria were initially evaluated using hematological parameters and serum ferritin. High-performance liquid chromatography (HPLC) was performed in participants with indices suggestive of β-thalassemia trait. As HPLC was not performed in all participants, this approach may introduce verification bias and potentially overestimate diagnostic accuracy. Those with abnormal chromatographic findings were counseled, and their spouses were advised to screen.

Outcome measures

All participants were followed until delivery. Maternal outcomes assessed included mode of delivery, preterm birth, intra-uterine growth restriction, placental abruption, need for blood transfusion, and pregnancy loss. Neonatal outcomes were assessed for all births, whereas specific variables, such as Apgar score and NICU admission, were analyzed only among live births. Neonatal outcomes included birth weight, need for neonatal intensive care unit admission, Apgar score at 5 minutes, neonatal anemia, requirement of neonatal transfusion, neonatal morbidity, and mortality. Missing data were minimal and handled by complete case analysis.

Data management and statistical analysis

Data collected were entered into Microsoft Excel (Microsoft Corporation, Redmond, USA) and analyzed using SPSS software version 27.0 (IBM Corp., Armonk, USA). Quantitative variables were expressed as mean and standard deviation, while qualitative variables were presented as frequencies and percentages. Comparison between groups for quantitative variables was performed using the unpaired t-test, and associations between categorical variables were assessed using the Chi-square test or Fisher’s exact test (where expected cell counts were <5), as appropriate. Confidence intervals were calculated for key diagnostic accuracy measures. The Pearson correlation coefficient was used to evaluate relationships between continuous variables. A p-value of less than 0.05 was considered statistically significant.

## Results

The majority of women in both groups were aged 26-30 years, comprising 76 (50.6%) in the iron deficiency anemia group and nine (42.9%) in the β-thalassemia trait group. This was followed by women aged >30 years, accounting for 38 (25.4%) and 6 (28.5%) respectively, and 20-25 years with 36 (24%) and 6 (28.6%) respectively. Mean age was comparable between groups (27.71 ± 3.61 vs 28.62 ± 4.73 years; p = 0.303). Mean BMI was significantly higher in the iron deficiency anemia group at 28.57 ± 3.36 compared to 26.05 ± 3.31 in the β-thalassemia trait group (p = 0.002). Most participants belonged to the lower middle socioeconomic class, accounting for 65 (43.3%) in the iron deficiency anemia group and 11 (52.4%) in the β-thalassemia trait group, followed by lower class with 48 (32%) and five (23.8%), with comparable distribution (p = 0.694). Hindus constituted the majority in both groups, with 94 (62.7%) in the iron deficiency anemia group and 13 (61.9%) in the β-thalassemia trait group, followed by Muslims with 37 (24.7%) and five (23.8%), respectively, with no significant difference in religious distribution (p = 0.109). Multigravida women predominated in both groups, comprising 99 (66%) in the iron deficiency anemia group and 12 (57.1%) in the β-thalassemia trait group, with comparable parity distribution (p = 0.796) (Table 1).

**Table 1:** Sociodemographic characteristics of pregnant women (n= 171).

Characteristics	Domain	Iron deficiency anemia (n = 150) n (%)	β-thalassemia trait (n= 21) n (%)	Test statistics
Age group	20-25 years	36 (24%)	6 (28.6%)	χ2= 0.4578 P= 0.7954
	26-30 years	76 (50.6%)	9 (42.9%)	
	>30 years	38 (25.4%)	6 (28.5%)	
Socioeconomic class	Lower	48 (32%)	5 (23.8%)	χ2= 1.447 P= 0.695
	Lower middle	65 (43.3%)	11 (52.4%)	
	Upper	3 (2%)	1 (4.8%)	
	Upper middle	34 (22.7%)	4 (19%)	
Religion	Hindu	94 (62.7%)	13 (61.9%)	χ2= 6.046 P= 0.109
	Muslim	37 (24.7%)	5 (23.8%)	
	Sikh	17 (11.3%)	1 (4.8%)	
	Christian	2 (1.3%)	2 (9.5%)	
Parity	Multigravida	99 (66%)	12 (57.1%)	χ2= 0.634 P= 0.796
	Primigravida	51 (34%)	9 (42.9%)	

Among 171 pregnant women with microcytic hypochromic anemia, iron deficiency anemia was observed in 150 (87.7%), while β thalassemia trait was identified in 21 (12.3%) participants. Within the β-thalassemia group, the majority were simple β-thalassemia carriers (15, 71.4%), followed by β-thalassemia with raised HbF (4, 19%), while β-thalassemia with hemoglobin D (HbD) trait and coexisting iron deficiency anemia were each seen in 1 (4.8%) participant. No cases of alpha thalassemia trait were detected (Table 2).

**Table 2:** Distribution of pregnant women with microcytic hypochromic anemia into iron deficiency anemia group and thalassemia sub groups (n= 171). HbD: Hemoglobin D; HbF: Fetal hemoglobin

Pregnant women with thalassemia carrier status/Iron deficiency anemia	Number (n = 171) n (%)
Iron deficiency anemia (IDA)	150 (87.7%)
β-thalassemia (thal) trait (BTT)	21 (12.3%)
β-thal trait	15 (71.4%)
β-thal trait + HbD trait	1 (4.8%)
β-thal trait + IDA	1 (4.8%)
β-thal trait + Raised HbF	4 (19%)
Alpha thalassemia trait	0

Mean hemoglobin and PCV were significantly lower in the β-thalassemia trait group (7.90 ± 1.36 g/dL and 25.42 ± 4.10%) compared to the iron deficiency anemia group (9.05 ± 0.93 g/dL and 27.55 ± 2.93%; p = 0.001 and 0.004, respectively). RBC count was significantly higher in the β-thalassemia trait group (5.19 ± 0.19 ×10⁶ cells per µl) than in the iron deficiency anemia group (4.44 ± 0.24 ×10⁶ cells per µl; p = 0.001). Red cell indices showed marked microcytosis and hypochromia in the β-thalassemia trait group, with significantly lower MCV (59.06 ± 4.81 fl vs 70.40 ± 3.37 fl) and MCH (18.07 ± 0.82 pg vs 23.30 ± 1.00 pg), while mean corpuscular hemoglobin concentration (MCHC) was slightly higher (29.24 ± 1.18 vs 28.49 ± 0.80; p = 0.001). RDW was also significantly lower in the β-thalassemia trait group (16.36 ± 0.51%) compared to the iron deficiency anemia group (17.13 ± 0.68%; p = 0.001). In the β-thalassemia trait group, mean HbA2 was 4.51 ± 0.64% and mean HbF was 2.25 ± 1.89% (Table 3).

**Table 3:** Hematological parameters (n= 171). RBC: Red blood cell; PCV: Packed cell volume; MCV: Mean corpuscular volume; MCH: Mean corpuscular hemoglobin; MCHC: Mean corpuscular hemoglobin concentration; RDW: Red cell distribution width; HbA2: Hemoglobin A2; HbF: Fetal hemoglobin

Hematological parameter	Iron deficiency anemia (n = 150) Mean ± SD	β-thalassemia trait (n= 21) Mean ± SD	Reference Range	P-value	t-value
Hemoglobin (g/dL)	9.05 ± 0.93	7.90 ± 1.36	11.0 – 15.0 (pregnancy-adjusted)	0.001	t = 3.76
PCV (%)	27.55 ± 2.93	25.42 ± 4.10	33.0 – 44.0	0.004	t = 2.30
RBC count (10^6 cells/ µl)	4.44 ± 0.24	5.19 ± 0.19	3.8 – 5.2	0.001	t = 16.40
MCV (fl)	70.40 ± 3.37	59.06 ± 4.81	80.0 – 100.0	0.001	t = 10.45
MCH (pg)	23.30 ± 1.00	18.07 ± 0.82	27.0 – 33.0	0.001	t = 26.60
MCHC (g/dL)	28.49 ± 0.80	29.24 ± 1.18	32.0 – 36.0	0.001	t = 2.82
RDW (%)	17.13 ± 0.68	16.36 ± 0.51	11.5 – 14.5	0.001	t = 6.19
HbA2 (%)	-	4.51 ± 0.64	2.0 – 3.5	-	-
HbF (%)	-	2.25 ± 1.89	< 1.0	-	-

All discriminant indices were significantly lower in the β thalassemia trait group compared to the iron deficiency anemia group. The mean Mentzer index was 11.38 ± 0.83, which was significantly lower in the β-thalassemia trait group versus 15.88 ± 1.10 in the iron deficiency anemia group (p = 0.001). Similarly, the Shine and Lal index was markedly lower in the β-thalassemia trait group (636.05 ± 116.47) compared to the iron deficiency anemia group (1158.67 ± 129.89; p = 0.001). The RDW index also showed significantly lower values in the β-thalassemia trait group (186.38 ± 16.31) than in the iron deficiency anemia group (272.40 ± 23.79; p = 0.001) (Table 4).

**Table 4:** Discriminant indices of iron deficiency anemia and β-thalassemia trait (n= 171).

Index	Iron deficiency anemia (n = 150) Mean ± SD	β-thalassemia trait (n= 21) Mean ± SD	P-value	t-value
Mentzer index	15.88 ± 1.10	11.38 ± 0.83	0.001	t = 22.30
Shine and Lal index	1158.67 ± 129.89	636.05 ± 116.47	0.001	t = 18.43
RDW index	272.40 ± 23.79	186.38 ± 16.31	0.001	t = 21.15

Receiver operating characteristic (ROC) analysis demonstrated excellent diagnostic performance of the indices. The Mentzer index, RDW index, and MCH showed perfect accuracy with an area under the curve (AUC) of 1.000, along with 100% sensitivity and 100% specificity (p = 0.001). The Shine and Lal index also showed near-perfect performance with an AUC of 0.996, sensitivity of 100%, and specificity of 95.2% (p = 0.001). MCV demonstrated high diagnostic accuracy with an AUC of 0.961, sensitivity of 98.7%, and specificity of 85.7% (p = 0.001). In contrast, RDW showed comparatively lower performance with an AUC of 0.818, sensitivity of 90.0%, and specificity of 47.6% (p = 0.001) (Table 5).

**Table 5:** Table showing comparative accuracy of indices of thalassemia (n= 171). AUC: Area under curve; CI: Confidence interval; ROC: Receiver operating characteristic; MCV: Mean corpuscular volume; MCH: Mean corpuscular hemoglobin; RDW: Red cell distribution width

Index	AUC	95% CI	Sensitivity (%)	Specificity (%)	p value (AUROC)
Mentzer Index	1.000	1.000 – 1.000	100	100	0.001
Shine and Lal Index	0.996	0.988 – 1.000	100	95.2	0.001
RDW Index	1.000	1.000 – 1.000	100	100	0.001
MCV (fL)	0.961	0.894 – 1.000	98.7	85.7	0.001
MCH (pg)	1.000	1.000 – 1.000	100	100	0.001
RDW (%)	0.818	0.726 – 0.910	90.0	47.6	0.001

Serum ferritin levels less than 15 ng per ml were observed in all women with iron deficiency anemia 150 (100%) compared to only one (4.8%) in the β-thalassemia trait group, showing a significant difference (p = 0.001). Conversely, ferritin levels greater than 15 ng per ml were seen in 20 (95.2%) women with β-thalassemia trait and none in the iron deficiency anemia group (Table 6).

**Table 6:** Serum ferritin levels in iron deficiency anemia and β-thalassemia trait groups (n= 171).

Serum ferritin	Iron deficiency anemia (n = 150) n (%)	β-thalassemia trait (n= 21) n (%)	Test statistics
<15 ng/ml	150 (100%)	1 (4.8%)	χ2=161.8 p=0.001
>15 ng/ml	0	20 (95.2%)	
Total	150 (100%)	21 (100%)	

Moderate anemia was the most common presentation in both groups, observed in 120 (80%) women with iron deficiency anemia and 13 (61.9%) with β-thalassemia trait. Mild anemia was more frequent in the iron deficiency anemia group - 28 (18.7%) - compared to one (4.8%) in the β thalassemia trait group. Severe anemia was significantly higher in the β-thalassemia trait group - seven (33.3%) - compared to two (1.3%) in the iron deficiency anemia group (p = 0.001) (Table 7).

**Table 7:** Severity of anemia in iron deficiency anemia and β-thalassemia trait groups (n= 171).

Severity of anemia	Iron deficiency anemia (n = 150) n (%)	β-thalassemia trait (n= 21) n (%)	Test statistics
Mild	28 (18.7%)	1 (4.8%)	χ2=38.72 p=0.001
Moderate	120 (80%)	13 (61.9%)	
Severe	2 (1.3%)	7 (33.3%)	
Total	150 (100%)	21 (100%)	

Iron transfusion was more commonly required in the iron deficiency anemia group - 33 (22%) - compared to one (4.7%) in the β-thalassemia trait group, with no statistically significant difference (p = 0.080). Packed red cell transfusion was significantly higher in the β-thalassemia trait group - 9 (42.9%) - compared to 20 (13.3%) in the iron deficiency anemia group (p = 0.003) (Table 8).

**Table 8:** Parenteral iron and packed red cells transfusion in iron deficiency anemia and β-thalassemia trait groups (n= 171).

Iron and packed red cells transfusion	Iron deficiency anemia (n = 150) n (%)	β-thalassemia trait (n= 21) n (%)	Test statistics
Iron transfusion	33 (22%)	1 (4.7%)	χ2=3.148 p=0.080
Packed red cells transfusion	20 (13.3%)	9 (42.9%)	χ2=5.467 p=0.003

Hypothyroidism was observed in 20 (13.3%) women with iron deficiency anemia and two (9.5%) with β-thalassemia trait (χ² = 0.238, p = 0.625), while gestational diabetes was seen in nine (6%) and two (9.5%), respectively (χ² = 0.380, p = 0.537), with no significant difference. Gestational hypertension was more frequent in the β-thalassemia trait group - three (14.3%) - compared to six (4%) in the iron deficiency anemia group (χ² = 3.909, p = 0.048). Preeclampsia was observed only in the β-thalassemia trait group - three (14.3%). Severe preeclampsia was significantly higher in the β-thalassemia trait group - two (9.5%) - compared to one (0.7%) in the iron deficiency anemia group (χ² = 8.384, p = 0.003). Antepartum hemorrhage was seen only in the iron deficiency anemia group - four (2.7%), while preterm premature rupture of membranes (PPROM) and oligohydramnios were slightly higher in the β-thalassemia trait group - one (4.8%) and two (9.5%) - compared to two (1.3%) and eight (5.3%), respectively (χ² = 1.256, p = 0.263 and χ² = 0.587, p = 0.443, respectively), with no significant difference (Table 9).

**Table 9:** Obstetric risk factors in iron deficiency anemia and β thalassemia trait groups (n= 171). HTN: Hypertension; APH: Antepartum hemorrhage; PPROM: Preterm premature rupture of membranes

Obstetric risk factors	Iron deficiency anemia (n = 150) n (%)	β-thalassemia trait (n= 21) n (%)	Test statistics
Hypothyroidism	20 (13.3%)	2 (9.5%)	χ2=0.238 p=0.625
Gestational diabetes	9 (6%)	2 (9.5%)	χ2=0.380 p=0.537
Chronic HTN	1 (0.7%)	0	NA
Gestational HTN	6 (4%)	3 (14.3%)	χ2=3.909 p=0.0480
Preeclampsia	0	3 (14.3%)	NA
Severe Preeclampsia	1 (0.7%)	2 (9.5%)	χ2=8.384 p=0.003
APH	4 (2.7%)	0	NA
PPROM	2 (1.3%)	1 (4.8%)	χ2=1.256 p=0.263
Oligohydramnios	8 (5.3%)	2 (9.5%)	χ2=0.587 p=0.443

There was attrition of three patients in the iron deficiency anemia group. Three patients in the iron deficiency anemia group were lost to follow-up, resulting in 168 evaluable cases for outcome analysis. Live birth was observed in 145 (98.6%) women with iron deficiency anemia and in 19 (90.5%) with β-thalassemia trait (χ² = 5.621, p = 0.076), while intrauterine fetal demise was higher in the β-thalassemia trait group - two (9.5%) - compared to two (1.4%) in the iron deficiency anemia group. Term vaginal delivery was significantly more common in the iron deficiency anemia group - 109 (74.1%) - compared to five (23.8%) in the β-thalassemia trait group, whereas preterm vaginal delivery (5, 23.8%), term lower segment cesarean section (LSCS) (7, 33.3%), and preterm LSCS (4, 19%) were higher in the β-thalassemia trait group (χ² = 27.05, p = 0.001). Premature rupture of membranes (PROM) was comparable between the groups (χ² = 0.098, p = 0.550), while complications such as Acute respiratory distress syndrome (ARDS) (1, 4.8%), pulmonary edema (1, 4.8%), and placental abruption (1, 4.8%) were observed only in the β-thalassemia trait group (χ² = 7.042, p = 0.008 for each).

Postpartum hemorrhage was significantly higher in the β-thalassemia trait group - three (14.3%) - compared to three (2%) in the iron deficiency anemia group (χ² = 8.000, p = 0.014), along with significantly higher ICU admission - two (9.5%) versus zero (χ² = 14.17, p = 0.015) - and significantly increased packed cell transfusion requirement - eight (38.1%) versus 17 (11.6%) (χ² = 10.21, p = 0.005). Iron transfusion was more common in the iron deficiency anemia group - 22 (15%) - compared to none in the β-thalassemia trait group (χ² = 3.616, p = 0.079). Uneventful outcomes were significantly higher in the iron deficiency anemia group - 91 (61.9%) - compared to one (4.8%) in the β-thalassemia trait group (χ² = 24.22, p = 0.001), with no maternal mortality in either group (Table 10).

**Table 10:** Maternal outcomes in iron deficiency anemia and β-thalassemia trait groups (n = 168). #Attrition of 3 patients in the iron deficiency anemia group. LSCS: Lower segment cesarean section; ARDS: Acute respiratory distress syndrome; PPH: Postpartum hemorrhage; ICU: Intensive care unit; PROM: Premature rupture of membranes

Maternal outcomes	Domain	Iron deficiency anemia (n = 147)^#^ n (%)	β-thalassemia trait (n= 21) n (%)	Test statistics
		145 (98.6%)	19 (90.5%)	χ2= 5.621 p=0.076
	Intrauterine fetal demise	2 (1.4%)	2 (9.5%)	
	Still born	0	0	
Mode of delivery	Term vaginal delivery	109 (74.1%)	5 (23.8%)	χ2= 27.05 p=0.001
	Preterm vaginal delivery	13 (8.8%)	5 (23.8%)	
	Term LSCS	22 (15%)	7 (33.3%)	
	Preterm LSCS	3 (2%)	4 (19%)	
PROM		5 (3.4%)	1 (4.8%)	χ2= 0.098 p=0.550
ARDS		0	1 (4.8%)	χ2= 7.042 p=0.008
PPH		3 (2%)	3 (14.3%)	χ2= 8.000 p=0.014
ICU admission		0	2 (9.5%)	χ2= 14.17 p=0.015
Packed cells transfusion		17 (11.6%)	8 (38.1%)	χ2= 10.21 p=0.005
Iron transfusion		22 (15%)	0	χ2= 3.616 p=0.079
Pulmonary edema		0	1 (4.8%)	χ2= 7.042 p=0.008
Placental abruption		0	1 (4.8%)	χ2= 7.042 p=0.008
Maternal mortality		0	0	-
Uneventful outcome		91 (61.9%)	1 (4.8%)	χ2= 24.22 p=0.001

Term births were significantly higher in the iron deficiency anemia group - 141 (95.9%) - compared to 13 (61.9%) in the β-thalassemia trait group, while preterm births were more frequent in the β-thalassemia trait group - eight (38.1%) versus six (4.1%) (χ² = 27.83, p = 0.001). Appropriate-for-gestational-age neonates were observed in 137 (93.1%) cases in the iron deficiency anemia group, significantly higher compared to nine (42.8%) in the β-thalassemia trait group, whereas small-for-gestational-age neonates were significantly higher in the β-thalassemia trait group - 12 (57.1%) versus 10 (6.8%) (χ² = 40.92, p = 0.001). Intrauterine growth restriction was significantly higher in the β-thalassemia trait group - 10 (47.6%) - compared to six (4.1%) in the iron deficiency anemia group (χ² = 40.42, p = 0.001). Normal birth weight was more common in the iron deficiency anemia group - 140 (95.2%) - compared to eight (38.1%) in the β-thalassemia trait group, while low birth weight neonates were significantly higher in the β-thalassemia trait group - 13 (61.9%) versus 7 (4.8%) (χ² = 57.21, p = 0.001).

Apgar score less than 7 at 5 minutes was higher in the β-thalassemia trait group - two (10.5%) - compared to two (1.3%) in the iron deficiency anemia group (χ² = 5.268, p = 0.076). Neonatal death occurred only in the β-thalassemia trait group - 1 (4.8%). NICU admission was significantly higher in the β-thalassemia trait group - 17 (89.4%) - compared to 23 (15.8%) in the iron deficiency anemia group (χ² = 43.20, p = 0.001). Respiratory distress was common in both groups - 11 (47.8%) versus eight (42.1%) (χ² = 17.17, p = 0.001), while other complications, including meconium aspiration syndrome (MAS) (χ² = 1.212, p = 0.331), neonatal hyperbilirubinemia (NNH) (χ² = 0.265, p = 0.487), sepsis (χ² = 5.268, p = 0.076), and transient tachypnea of newborn (TTN) (χ² = 2.469, p = 0.164) were comparable. Hydrocephalus, neonatal anemia, and neonatal transfusion were observed only in the β-thalassemia trait group - one (5.2%) each (χ² = 7.042, p = 0.008 for each) (Table 11).

**Table 11:** Neonatal outcomes in iron deficiency anemia group and β-thalassemia trait (n=168). ^Only live birth considered (n = 145 in IDA group, n = 19 in BTT group); ^#^Attrition of 3 patients in iron deficiency anemia group. AGA: Appropriate for gestational age; SGA: Small for gestational age; IUGR: Intrauterine growth restriction; NICU: Neonatal intensive care unit; RD: Respiratory distress; MAS: Meconium aspiration syndrome; NNH: Neonatal hyperbilirubinemia; TTN: Transient tachypnea of newborn; IDA: Iron deficiency anemia; BTT: β-thalassemia trait

Neonatal outcomes	Iron deficiency anemia (n = 147)^#^ n (%)	β-thalassemia trait (n= 21) n (%)	P-value (Chi-Square)
Term birth	141 (95.9%)	13 (61.9%)	χ2= 27.83 p=0.001
Preterm birth	6 (4.1%)	8 (38.1%)	
AGA	137 (93.1%)	9 (42.8%)	χ2= 40.92 p=0.001
SGA	10 (6.8%)	12 (57.1%)	
IUGR	6 (4.1%)	10 (47.6%)	χ2= 40.42 p=0.001
Birth weight (>2500 gm )	140 (95.2%)	8 (38.1%)	χ2= 57.21 p=0.001
Low birth weight (<2500 gm)	7 (4.8%)	13 (61.9%)	
Apgar < 7 at 5 minutes^	2 (1.3%)	2 (10.5%)	χ2= 5.268 p=0.076
Neonatal death	0	1 (4.8%)	NA
NICU admission^	23 (15.8%)	17 (89.4%)	χ2= 43.20 p=0.001
RD	11 (47.8%)	8 (42.1%)	χ2= 17.17 p=0.001
MAS	2 (8.6%)	1 (5.2%)	χ2= 1.212 p=0.331
NNH	4 (17.3%)	1 (5.2%)	χ2= 0.2650 p=0.487
Sepsis	2 (8.6%)	2 (10.5%)	χ2= 5.268 p=0.076
TTN	4 (17.3%)	2 (10.5%)	χ2= 2.469 p=0.164
Hydrocephalus	0	1 (5.2%)	χ2= 7.042 p=0.008
Neonatal anaemia	0	1 (5.2%)	χ2= 7.042 p=0.008
Neonatal transfusion	0	1 (5.2%)	χ2= 7.042 p=0.008

## Discussion

The present prospective hospital-based study evaluated pregnant women with microcytic hypochromic anemia to differentiate iron deficiency anemia from β-thalassemia trait and to assess associated maternal and neonatal outcomes. A total of 171 women were included, of whom 87.7% had iron deficiency anemia, and 12.3% had β-thalassemia trait. The majority of participants belonged to the 26 to 30-year age group, consistent with findings by Chaudhary et al. [16]. Body mass index was significantly higher in the iron deficiency anemia group, in contrast to Dasgupta et al., who reported comparable values between groups. No significant differences were observed in parity, socioeconomic status, or religion, similar to a previous study [17].

Among β-thalassemia cases, most were simple carriers, with a smaller proportion showing raised HbF or coexisting hemoglobin variants such as HbD Punjab. This aligns with studies by Sur and Chakravorty and Swaroop et al., which also reported β-thalassemia trait as the most common hemoglobinopathy [18, 19]. The presence of raised HbF may be attributed to co-inheritance of modifying polymorphisms, while coexistence with iron deficiency anemia has been previously described by Verma et al. [20].

Hematologically, β-thalassemia trait was associated with significantly lower hemoglobin, hematocrit, MCV, and MCH values compared to iron deficiency anemia. These findings are consistent with those of Ruangvutilert et al. and Dasgupta et al. [2, 17]. Red cell indices demonstrated marked microcytosis and hypochromia, reinforcing their diagnostic utility. In the present study, MCH and MCV showed strong predictive value, in agreement with Mayuri et al., who identified these indices as reliable markers for β-thalassemia screening [21].

Discriminant indices such as the Mentzer index, the Shine and Lal index, and the RDW index showed excellent diagnostic performance. The Mentzer index demonstrated perfect sensitivity and specificity, consistent with prior literature [22], although other studies have reported slightly lower sensitivity [1]. The Shine and Lal, and RDW indices also showed near-perfect accuracy, supporting their use as adjunct tools. Similar findings were reported by Mayuri et al., who demonstrated high diagnostic accuracy for multiple indices [21]. These results emphasize that combining multiple indices improves screening accuracy rather than relying on a single parameter.

Severity of anemia was greater in the β-thalassemia trait group, with a higher proportion of severe anemia compared to iron deficiency anemia. This may be explained by ineffective erythropoiesis and chronic hemolysis in thalassemia [23]. Comparable trends were reported by Swaroop et al. and Mishra et al., where moderate anemia was most common, but severe anemia was also observed [19, 24].

Iron therapy was primarily required in the iron deficiency anemia group, whereas transfusion requirements were significantly higher in the β-thalassemia trait group. These findings are consistent with Berwal et al., who reported increased transfusion needs and maternal complications in thalassemic pregnancies [25]. Serum ferritin levels effectively distinguished between the two conditions, with low levels seen in all iron deficiency anemia cases and rarely in β-thalassemia trait. Improvement in hemoglobin following iron therapy in a patient with coexisting deficiency supports findings by Chen et al., highlighting the importance of identifying concurrent iron deficiency [26].

Hypertensive disorders, including gestational hypertension and severe preeclampsia, were more frequent in the β-thalassemia trait group, in line with Ruangvutilert et al. [2]. This may be related to increased oxidative stress and inflammatory cytokines, leading to placental dysfunction. Obstetric outcomes also differed significantly, with higher rates of cesarean delivery, postpartum hemorrhage, and transfusion requirement in the β-thalassemia group, consistent with studies by Amooee et al. and Dasgupta et al. [11, 17]. Severe complications such as ARDS and ICU admission were observed only in the β-thalassemia group, although no maternal mortality occurred.

Adverse neonatal outcomes were markedly higher among β-thalassemia carriers. Preterm birth, intrauterine growth restriction, low birth weight, and NICU admissions were significantly more common, consistent with findings by Mayuri et al., Goswami et al., and St-George's et al. [21, 27, 28]. These outcomes are likely related to chronic maternal anemia and placental insufficiency.

The strengths of this study include its prospective design, use of HPLC for confirmation, and evaluation of simple red cell indices and multiple discriminant indices, including less commonly used parameters such as the Shine and Lal index, with serum ferritin estimation proving highly effective in differentiating iron deficiency anemia from β-thalassemia trait. However, limitations include its single-center design, relatively small number of β-thalassemia cases, and restriction to women with microcytic hypochromic anemia, which may have excluded carriers with normal indices. Additionally, no single index demonstrated universal applicability, as atypical presentations may occur due to genetic interactions or mild mutations.

Overall, this study underscores the utility of simple hematological indices as cost-effective preliminary screening tools for β-thalassemia trait in resource-limited settings; however, confirmatory testing with HPLC remains essential. The findings should be interpreted in light of several limitations. This was a single-center study, which may limit generalizability. The relatively small number of β-thalassemia trait cases (n=21) reduces statistical power and may lead to unstable estimates, particularly for less frequent outcomes. Selection bias is also likely, as only pregnant women with microcytic hypochromic anemia were included. In addition, not all participants underwent confirmatory HPLC testing, as it was primarily performed in those with indices suggestive of β-thalassemia trait; this may introduce verification bias and potentially overestimate diagnostic accuracy.

Serum ferritin was used both in initial classification and subsequent evaluation, which introduces the possibility of circular reasoning and warrants cautious interpretation. Furthermore, outcome analyses were unadjusted, and potential confounders such as the severity of anemia, BMI, and associated comorbidities were not controlled. Some subgroup comparisons also involved small numbers and should therefore be interpreted cautiously. Future large-scale, multicentric studies incorporating universal confirmatory testing, molecular diagnostics, and long-term follow-up are needed to validate these findings and strengthen screening strategies for improved maternal and neonatal outcomes.

## Conclusions

Among pregnant women with microcytic hypochromic anemia in this single-center cohort, β-thalassemia trait was identified in 12.3% of the participants and was associated with more severe anemia and adverse maternal and neonatal outcomes. Red cell indices combined with serum ferritin may provide useful preliminary screening information; however, confirmatory testing with HPLC remains essential. Larger multicenter studies with universal confirmatory testing and adjusted analyses are required to validate these findings.
